# Emerging Roles for Glycogen in the CNS

**DOI:** 10.3389/fnmol.2017.00073

**Published:** 2017-03-16

**Authors:** Alice E. Waitt, Liam Reed, Bruce R. Ransom, Angus M. Brown

**Affiliations:** ^1^School of Life Sciences, University of NottinghamNottingham, UK; ^2^Department of Neurology, University of WashingtonSeattle, WA, USA

**Keywords:** glycogen, glucose, lactate, astrocyte, potassium, memory, aglycemia, exercise

## Abstract

The ability of glycogen, the depot into which excess glucose is stored in mammals, to act as a source of rapidly available energy substrate, has been exploited by several organs for both general and local advantage. The liver, expressing the highest concentration of glycogen maintains systemic normoglycemia ensuring the brain receives a supply of glucose in excess of demand. However the brain also contains glycogen, although its role is more specialized. Brain glycogen is located exclusively in astrocytes in the adult, with the exception of pathological conditions, thus in order to benefit neurons, and energy conduit (lactate) is trafficked inter-cellularly. Such a complex scheme requires cell type specific expression of a variety of metabolic enzymes and transporters. Glycogen supports neural elements during withdrawal of glucose, but once the limited buffer of glycogen is exhausted neural function fails and irreversible injury ensues. Under physiological conditions glycogen acts to provide supplemental substrates when ambient glucose is unable to support function during increased energy demand. Glycogen also supports learning and memory where it provides lactate to neurons during the conditioning phase of *in vitro* long-term potentiation (LTP), an experimental correlate of learning. Inhibiting the breakdown of glycogen or intercellular transport of lactate in *in vivo* rat models inhibits the retention of memory. Our current understanding of the importance of brain glycogen is expanding to encompass roles that are fundamental to higher brain function.

## Introduction

The lack of appreciation for the role(s) that brain glycogen contributes to brain function cannot be attributed to its recent discovery, as its presence, detected by both biochemical assay (Koizumi, 1974) and electron microscopy (Koizumi and Shiraishi, 1970), has been known for decades. Unlike skeletal muscle or liver, where glycogen is expressed uniformly throughout a homogenous cell population (Stryer, 1995), the cellular (and sub-cellular) location of glycogen within the brain provides clues to its function (Cataldo and Broadwell, 1986; Oe et al., 2016), but detailed information regarding the intricacies of metabolic cell-to-cell signaling within the brain (Dringen et al., 1995; Magistretti and Pellerin, 1997; Dienel, 2009) were required before potential roles for glycogen could be proposed and experimentally tested (Brown et al., 2003; Suzuki et al., 2011). This disregard for brain glycogen was compounded by the low concentration with which glycogen is expressed in the brain; up to 100 times lower than liver and skeletal muscle (Stryer, 1995). Although never consigned as a vestigial entity glycogen was quietly ignored, and in a neurocentric world there were few advocates proposing it warranted detailed investigation.

## Systemic Hypoglycemia

**Figure 1:**
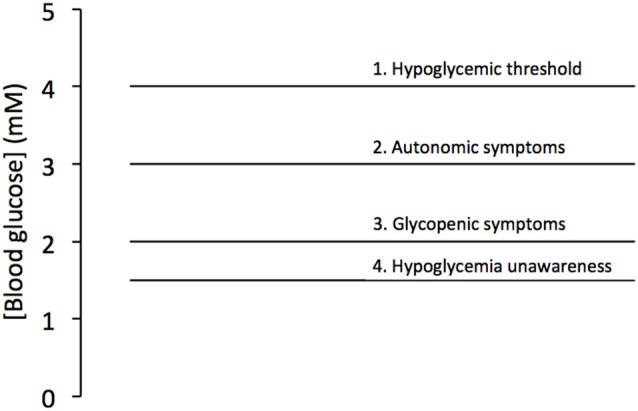
**Blood glucose levels associated with hypoglycemia.** Blood glucose below 4 mM is considered hypoglycemic (1), with autonomic symptoms (2) triggered at about 3 mM and glycopenic symptoms triggered at about 2 mM (3). However the condition hypoglycemia unawareness leads to a fall in the threshold of the autonomic symptoms below that of the glycopenic threshold (4). Adapted from Figure 7.1 (Frier and Fisher, 2007).

The presence of glycogen in the brain may be viewed as at odds with established facts regarding whole body metabolism. Given the brains exclusive reliance on glucose as its sole energy support, and the complex endocrine machinery that secures blood glucose levels within a narrow concentration range (Frier and Fisher, 2007), any need for glycogen within the brain initially seems superfluous. The liver breaks down glycogen in response to falling systemic glucose levels ensuring adequate delivery of glucose to the brain for up to 24 h (Stryer, 1995). Skeletal muscle glycogen provides a localized energy reserve for muscle (Champe and Harvey, 2008; although even this is a simplification, with recent studies suggesting a highly complex and coordinated reciprocal metabolism of glucose and lactate between brain and muscle, dependent upon immediate localized requirements; Dalsgaard et al., 2004; Dalsgaard, 2006). What is not in doubt is the exquisite sensitivity that the brain displays when confronted with a shortfall in glucose delivery (Frier and Fisher, 2007). Consistent with the homeostatic hormonal response that maintains normoglycemia, in event of decreased systemic glucose (hypoglycemia) the brain responds via the autonomic nervous system, with a series of signals (sweating, tremor, decreased temperature, decreased intraocular pressure, increased gastric emptying) that warn the sufferer of an impending hypoglycemic attack (Frier and Fisher, 2007). The critical aspect of this autonomic response is that its threshold lies above that of the glycopenic symptoms (confusion, drowsiness, odd behavior, speech difficulty, lack of co-ordination) of hypoglycemia characterized by confusion. However repeated hypoglycemic episodes compromise the threshold for the autonomic symptoms, a process called hypoglycemia unawareness (Cryer, 2002), such that they dip below those of the glycopenic effects rendering the sufferer incapable of responding appropriately to the hypoglycemia, with potentially fatal consequences (Figure 1).

## Glucose Is the Main Energy Support for Brain Function

In the last 20 years attempts to deduce the role(s) of brain glycogen have been played out against a background of confusion, dead ends and polarized opinion, which stems from the simple fact that we know little about critical aspects of metabolism in the brain at the cellular level (Ames, 2000). The scenario can be simplified as follows; we do not know which energy substrates cells use under particular conditions. This rather simplistic statement conceals a degree of complexity that is initially difficult to appreciate when one considers that all we are considering is which of two energy substrates (glucose or lactate) two cell types (neurons and glia) use. To begin to unravel this, we must first review evidence that led to the dogmatic view of brain energy metabolism, which states that glucose is the energy substrate used by all cells in the brain (Dwyer, 2002). This has substantial support based on the following facts: (1) during insulin-induced hypoglycemia there is no systemic alternate energy substrate that can substitute for glucose; (2) complex homeostatic mechanisms have evolved to maintain normoglycemia (no such equivalent systems exist for alternate substrates such as lactate); (3) non-glucose substrates are converted, in the process of gluconeogenesis, into glucose, implying glucose is a universally preferred substrate; (4) metabolites of glucose are detected in the brain using NMR spectroscopic methods; and (5) the arterial-venous difference for brain glucose is positive, implying glucose uptake into the brain. Although it has been known for decades that the brain releases lactate (Abi-Saab et al., 2002), historically this was considered a waste product of glucose metabolism, and of no metabolic consequence. This dismissal of lactate as a viable energy substrate was reconsidered in the face of evidence that suggested it might be actively taken up and metabolized by neurons. However the conditions under which this would occur were obscured due to the technical difficulties involved in measuring energy usage in single cells. An additional complication is the role that glycogen plays. Glycogen, the storage form of glucose in the body (Champe and Harvey, 2008), is a molecule comprising dehydrated glucosyl molecules, which can be liberated as lactate (Shulman et al., 2001).

## Sub-Cellular Location of Glycogen

Since the advent of the electron microscope it has been known that in the adult rodent brain glycogen is stored almost exclusively in astrocytes (Cataldo and Broadwell, 1986). Due to a lack of appreciation of the role that glial cells contribute to brain function that was prevalent in previous decades, such a location for glycogen, compounded by its low concentrations, led to dismissal of any importance attached to the compound (Stryer, 1995). We should not judge our predecessors for their lack of insight, as it is only in recent years, where the intercellular metabolic signaling pathways have been identified, that a role for glycogen has become apparent (Brown et al., 2003; Suzuki et al., 2011). Initial studies, which reported that glycogen was located in astrocyte processes abutting synapses (Koizumi and Shiraishi, 1970; Phelps, 1972; Koizumi, 1974), have been confirmed with more advanced microscopic techniques that allow 3D reconstruction of very fine astrocytic processes (Cali et al., 2016). These studies suggest not only an intimate relationship exists between astrocytes and neurons, but in particular between astrocytes and synapses, with mitochondria expressed in high density in these processes (Cali et al., 2016).

Until recently the regional expression of brain glycogen was unclear. This was primarily because the technique used to assess glycogen expression (biochemical glycogen assay) was incapable of the resolution required to accurately measure the glycogen concentration in small areas of brain (Passonneau et al., 1967). A refined technique that uses immunohistochemical assessment of glycogen has recently been used to accurately visualize glycogen expression throughout the rodent brain (Oe et al., 2016). The report confirmed that in the adult glycogen is expressed almost exclusively in astrocytes and is located in the astrocytic processes rather than in the soma. The highest expression of glycogen occurred in the hippocampus, striatum, cortex and cerebellar molecular layer, a region of high synaptic activity, but was lower in white matter and sub-cortical areas. It is interesting to note that the regions with the highest expression of glycogen also have higher metabolic demand.

The dogmatic view of an exclusively astrocytic domain for glycogen must be considered against the evidence of neuronally located glycogen. Such a location was associated with pathological conditions such as Lafora’s disease, a form of epilepsy, where such an over-abundance of neuronal glycogen is considered a side effect of the disease and has no physiological function (Vilchez et al., 2007). In the sciatic nerve glycogen related enzymes are shown to be present in axons (Pfeiffer-Guglielmi et al., 2006, 2007), and glycogen is present in both axons and Schwann cells, but only under pathological conditions (Powell et al., 1985; Katsuragi et al., 1988; Kalichman et al., 1998). A recent article has described neuronal glycogen that acts to protect axons during periods of hypoxia, as glycogen phosphorylase (GP) is present in nerves facilitating glycogen metabolism (Saez et al., 2014).

## Astrocytic Support of Cultured Neurons

Initial studies that investigated the role of neuronal cells were carried out in tissue culture conditions. This reductionist approach was justified in the light of the difficulty of gaining meaningful data from *in situ* tissue (Kettenmann and Grantyn, 1992). These studies yielded bountiful information, but it was only as the studies progressed that questions were raised as to their validity i.e., did what occurred in the artificial nature of the tissue culture dish have any relation to what occurred *in vivo*. A key example of this was the viability of neurones in culture, where the yield of neurones cultured in isolation was poor. As the neurones cultured were from neo-natal animals, these neurones had to develop in the absence of any support from the *in vivo* environment they would normally develop in. Glial cells were added to the culture in an attempt to increase neurone yield, which did indeed occur although the reasons were unknown (Whatley et al., 1981). We now know that neurones are reliant on glial cells for a multitude of reasons; release of trophic factors to guide developing neurones, to provide myelin, to buffer extracellular compounds such as potassium and glutamate. In addition the extracellular space is essentially infinite in culture conditions, whereas *in vivo* it is relatively small (15% of brain volume) and provides a very small buffer zone between neighboring cells (Swanson and Choi, 1993). However studies using tissue culture from the Waagepetersen laboratory confirm that glia-neuron transfer of metabolites interactions can occur (Sickmann et al., 2005). The glia and other surrounding neurones would also provide structural support to maintain the neuron in a fixed location within the brain parenchyma. Although not the focus of this review, oligodendrocytes are implied to play a role in supplying lactate to axons (Lee et al., 2012).

Whereas the behavior of neurones may have been compromised in tissue culture conditions, it was a serendipitous approach that yielded preliminary information on the metabolic support afforded neurones by astrocytes (Swanson and Choi, 1993). Neurones that were co-cultured with astrocytes displayed increased viability compared to neurones that were cultured in isolation. However it was discovered that it was the presence of glycogen in the astrocytes that was the critical factor in promoting neuronal survival, as astrocytes with depleted glycogen did not significantly prolong neuronal survival (Swanson and Choi, 1993). This was an intriguing result that posed more questions than it answered, most notably, what was the form in which glycogen provided support?

## The Role of Glycogen during Aglycemia

From studies of glycogen in the liver and skeletal muscle the nature of the glycogen molecule and its regulation are well established (Stryer, 1995), information that can reasonably be applied to brain glycogen. In the periphery glycogen acts as a storage depot for excess glucose during periods of plenty, to be liberated in time of need. In the liver glycogen is liberated in the form of glucose to maintain normoglycemic blood glucose levels (Stryer, 1995), whereas in the muscle glycogen is glycolytically metabolized (Stryer, 1995), with the apparent waste product lactate liberated into the bloodstream. The nature of the support offered by astrocytic glycogen to neurons was presumably metabolic, thus how this was achieved was a prime focus of investigation. The simplest explanation was that glycogen supported solely astrocytes, relieving the metabolic burden from neurones, i.e., astrocytes are fueled exclusively be glycogen sparing interstitial glucose for exclusive use by neurons (DiNuzzo et al., 2010). Whilst this is a theory as simplistic as it is attractive, it is not the case. Studies carried out in cultured astrocytes indicated that when astrocytes were incubated in the presence of glucose they released lactate (Dringen et al., 1993b), whereas neurones did not release lactate; in fact they consumed it (Dringen et al., 1993a). This indicated that astrocytes might release lactate, which is then taken up and used by neurons as an energy substrate to supplement glucose uptake (Dringen et al., 1993a). This is where the origins of a contentious and continuing dispute arose. Whilst it can be insightful to study how processes work under tissue culture conditions, they may not apply *in vivo*. This is especially important where the interstitial compartment is proposed to be the medium of exchange through which putative metabolic compounds are passed from astrocyte to neurons. However under tissue culture conditions where the interstitial space is infinitely large, this is unrealistic and alternate models were required.

**Figure 2:**
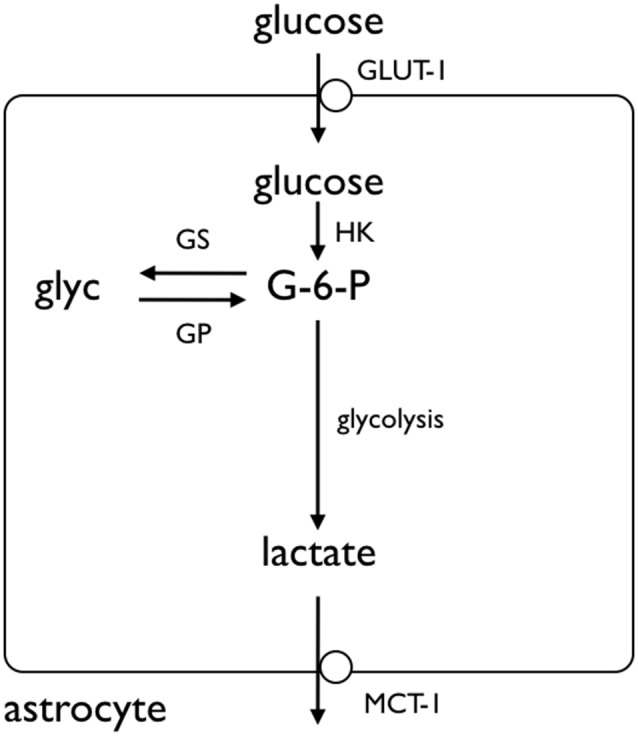
**Glycogen storage in the astrocyte.** Glucose is taken up by astrocytes via the GLUT-1 glucose transporter, where it is phosphorylated by hexokinase (HK) to glucose-6-phosphate (G-6-P). The G-6-P can either proceed along the glycolytic pathway, or be added to the glycogen molecule by glycogen synthase (GS). The glycogen is broken down by glycogen phosphorylase (GP), ultimately to lactate, which crosses the astrocyte membrane by monocarboxylate transporter (MCT). Adapted from Figure 1 (Fryer and Brown, 2015).

Given the complexity of metabolic interactions in gray matter the Ransom lab chose to use a simple white matter model, the rodent optic nerve, to investigate the role of glycogen (Ransom et al., 1994). These studies were prompted by comparison of the effects of anoxia or aglycemia on the stimulus evoked compound action potential (CAP). The tissue is accessible to recordings of the stimulus evoked CAP, a monitor of axon conduction, whose area is proportional to the number of conducting axons (Cummins et al., 1979). The CAP area recorded in 10 mM glucose provides a baseline measure of axon conduction against which the post-insult CAP can be compared (Stys et al., 1991). In addition to electrophysiological recordings, the tissue is amenable to biochemical assay, simultaneous recordings of lactate release from the tissue and immunohistochemical staining. In the presence of oxygen but with glucose withdrawn (aglycemia) the CAP lasted up to 30 min before failing (Ransom and Fern, 1997). This suggested that there was an endogenous energy reserve present within the tissue that supported axon conduction in the absence of exogenously applied glucose, but that the reserve was limited and could only support conduction for a limited period of time; once the reserve was exhausted conduction failed. The most likely candidate for this energy reserve is glycogen, which has been located in astrocytes by electron microscopy in both the rat (Wender et al., 2000) and mouse optic nerve (MON; Brown et al., 2003). Given that glycogen supports axon conduction in the absence of glucose, one would reasonably predict that increasing glycogen content should increase the latency to CAP failure, whereas depleting glycogen content would attenuate latency to CAP failure, during a subsequent period of aglycemia. This was indeed found to be the case (Brown et al., 2003). The macromolecule glycogen is too large to be transported intercellularly, thus there must exist a glycogen-derived metabolic conduit that supports axon conduction. This is most likely lactate, since astrocytes in culture release lactate in the presence of glucose (Dringen et al., 1993a,b). If lactate were released from the breakdown of glycogen, then lactate, in the absence of glucose, should be able to support MON axon conduction. In addition, blocking lactate release from astrocytes, or uptake into axons with pharmacological blockers should also attenuate the CAP: all of these predictions were shown to occur experimentally (Brown et al., 2003; Tekkok et al., 2005). Immunohistochemical studies to investigate the presence of monocarboxylate transporters (MCTs) on cells membranes, demonstrated MCT1 immunoreactivity on astrocyte membranes and MCT2 immunoreactivity on axon membranes (Tekkok et al., 2005). The MCTs facilitate the movement of lactate across cell membranes, thus their presence is an absolute requirement for trans-membrane flux of lactate. Preventing glycogen breakdown by pharmacologically inhibiting GP also attenuated the latency to CAP failure during aglycemia (Brown et al., 2004; Figure 2).

**Figure 3:**
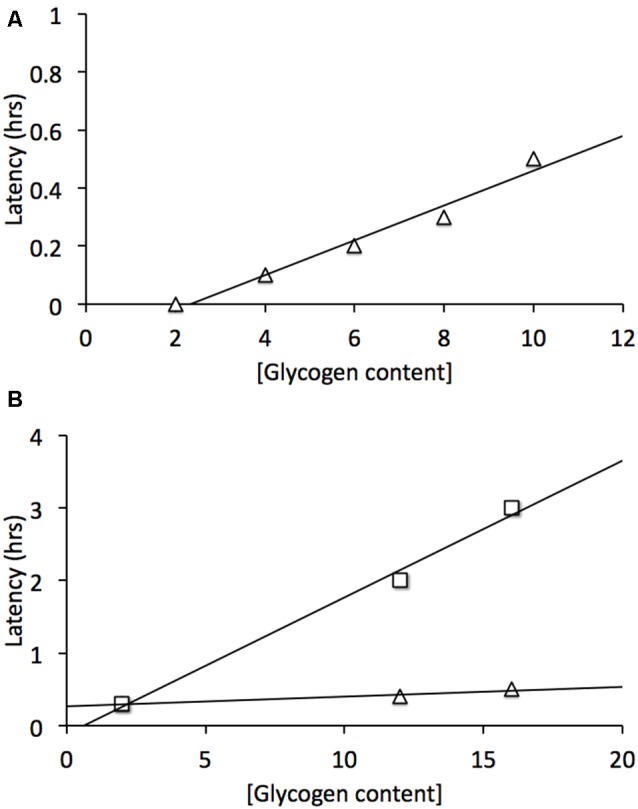
**Glycogen content determines latency to failure in optic nerve axons and Schwann cell A fibers but not C fibers. (A)** In mouse optic nerve (MON) glycogen content determines latency to failure during subsequent period of aglycemia (Δ). **(B)** In Schwann cells elevated glycogen content benefits A fibers (□) but not C fibers (Δ). Note elevated (glycogen) does not prolong C fiber latency to failure. Adapted from Figure 2 (Brown et al., 2003) and Figure 5 (Brown et al., 2012).

The sciatic nerve was studied in order to determine whether there was an equivalent metabolic cell-to-cell signaling pathway in the peripheral nervous system (PNS). Electron microscopic images revealed that Schwann cells did express glycogen, which was measured by biochemical assay at a concentration about twice that in the optic nerve (Brown et al., 2012). The sciatic nerve contains two broad classes of axons, large myelinated A fibers, and smaller unmyelinated C fibers grouped within Remak bundles (Landon, 1976). Introduction of aglycemia resulted in the C peak failing within about 30 min, whereas the larger myelinated A fibers could sustain conduction for up to two and a half hours before they began to fail (Brown et al., 2012). Such a difference could in part be related to the greater metabolic rate of small axons (per unit volume) compared to larger axons (Perge et al., 2012). However when glycogen content was increased there was no increase in the latency to failure in the C peak, whereas the latency to failure of A fibers was increased (Figure 3). Thus glycogen expressed in Schwann cells only benefitted the A fibers and had no effect on the C fibers. Glycogen was broken down to lactate in the Schwann cell and transported intercellularly via MCTs (Domènech-Estévez et al., 2015) to the A fibers (Brown et al., 2012). Thus, there are striking similarities between the central and PNSs with regard to the metabolic provision of glycogen-derived lactate, where glycogen, located in glial cells, is broken down to lactate, which acts as a metabolic conduit to support axon conduction during periods of shortfall in glucose delivery to the tissue. However what is more physiologically relevant is the role(s) that glycogen plays under normal, non-pathological conditions, thus the next logical step in the MON studies was to investigate the role of glycogen in supporting physiological activity.

## The Role of Glycogen during High Frequency Stimulus

Imposing a short period of high intensity stimulus was very effective at revealing key information. At frequencies between 10 Hz and 100 Hz, i.e., an action potential was evoked between every 100 ms and 10 ms, the increasing metabolic demand imposed on the tissue exceeded its ability to support conduction, in the presence of 10 mM glucose aCSF (Brown et al., 2003). In the first instance imposing a 4 min period of 100 Hz stimulus on the MON caused an elongation of the CAP profile, no doubt due to accumulation of interstitial K^+^ (Connors et al., 1982) exceeding the ability of the Na^+^-K^+^ ATPase to maintain homeostatic trans-membrane ion concentrations (Ransom et al., 2000), but the CAP area did not fall below baseline (Brown et al., 2003). However a decrease in glycogen content after only 4 min of the high intensity stimulus occurred in the presence of 10 mM glucose (Brown et al., 2003), suggesting that even this high concentration of glucose was unable to support the CAP, and glycogen derived lactate was released as a supplemental energy substrate. Extended exposure to 100 Hz stimulus (on the order or tens of minutes) caused a decrease in the CAP area that could be reversed if the MON was supplied with 30 mM glucose aCSF (Brown et al., 2003). This reveals a very important point, which is that the MON will continue to conduct action potentials during periods of increased energy demand if excess substrate is applied, i.e., if the increased energy demand required by the increased stimulus is met by increased supply of energy substrate then CAP conduction will be maintained. Thus, there is no absolute threshold with regard to conduction failure; rather it is determined on the basis of whether the energy demand is matched by supply of substrate. This point was further demonstrated by exposing MONs to 2 mM glucose aCSF. Under baseline conditions of stimulus every 30 s the CAP area was maintained at baseline levels. However depleting glycogen with a short period of exposure to 0 mM glucose resulted in subsequent exposure to 2 mM glucose being unable to support conduction (Brown et al., 2003), implying that under hypoglycemic conditions supplementary glycogen-derived lactate is required to fully support function. The relationship between the ability of the MON to support conduction under the duress of increased energy demand was shown when MONs were exposed to 100 Hz stimulus in 2 mM glucose, where the CAP fell rapidly on imposition of the stimulus (Brown et al., 2003). These experiments provided the first information regarding potential role(s) of glycogen under physiological conditions, namely to act as an energy buffer to supply substrate during short-term mis-match between ambient glucose and energy demand.

A key issue that was missing from these studies was identification of the signaling mechanism whereby neurones inform astrocytes of their metabolic requirements. Such a mechanism would have to adhere to the following requirements: (1) it must be localized; (2) it must be capable of invoking rapid glycogen breakdown; (3) the intensity of the signal must be proportionate to the stimulus intensity; (4) the intensity of the signal must dissipate as the stimulus intensity declines; and (5) if the signal is not universal, then comparable localized equivalent systems must exist across the nervous system. A potential mechanism was revealed via studies investigating the neurotransmitter modulation of glycogen content.

## The Astrocyte Neuron Lactate Shuttle Hypothesis

One important aspect of glycogen’s metabolism in the brain is the means by which it is modified. Whereas in the periphery glycogen is modulated by the hormones insulin and glucagon, in the brain parenchyma the influence of these hormones appear to be attenuated and neurotransmitters contribute regulatory roles. Pierre Magistretti’s group started investigating this phenomenon in the early 1990’s. Their goal was to identify physiologically relevant modulators, and these initial studies identified VIP, nor-epinephrine and adenosine as well as K^+^ (see later) as controlling glycogen levels (Hof et al., 1988; Sorg and Magistretti, 1991). However their studies investigating the putative modulatory role of the most abundant excitatory CNS neurotransmitter, glutamate, yielded unexpected results that were adapted as the basis of the astrocyte neuron lactate shuttle hypothesis (ANLSH; Pellerin and Magistretti, 1994). This hypothesis had its origins in studies carried out in the honeybee retina, which is metabolically and morphologically compartmentalized. Light stimulus induces glucose uptake in the honeybee retinal glial cells, but these cells show no increased oxygen uptake. However the photoreceptors take up alanine derived from the astrocytic glucose and increase oxygen consumption (Tsacopoulos et al., 1994). This metabolic compartmentalization clearly displays a division of labor between glial cells and neurons, in which the glial cells take up glucose and glycolytically convert it to alanine, which is then transported to the photoreceptors via the interstitial space for oxidative metabolism. Magistretti and Pellerin (1997) work proposed a similar compartmentalized scenario in the mammalian brain, which is set in motion when neuronal activity results in elevated interstitial glutamate. The glutamate is co-transported with Na^+^ into the astrocyte, where the glutamate is converted to glutamine, then shuttled to the neurones for conversion to glutamate by glutaminase. However the excess Na^+^ must be pumped out of the astrocyte via the Na^+^-K^+^ ATPase and conversion of glutamate to glutamine requires ATP. The energy debt is met via astrocytic uptake of glucose followed by glycolytic conversion to lactate, yielding two molecules of ATP. One molecule of ATP fuels the Na^+^-K^+^ ATPase, the other glutamate conversion. The product of the glycolysis, lactate, is then transported out of the astrocyte and into the neurone where it is oxidatively metabolized (Pellerin and Magistretti, 1994). This is a superficially stoichiometrically pleasing scheme but has come in for intense scrutiny, key criticisms being that this scheme obviously only applies to glutamatergic brain areas; what occurs, for example, in white matter, and if all glucose and glycogen goes to lactate what fuels other processes? The debate is too convoluted and voluminous to describe in detail here, but the following reviews may be consulted to provide a balanced view of both sides of the argument with those opposed to the hypothesis proposing arguments that cannot be ignored (Chih et al., 2001; Dienel and Hertz, 2001; Chih and Roberts, 2003; Pellerin and Magistretti, 2003, 2012; Aubert et al., 2005; Dienel, 2010; DiNuzzo et al., 2010).

## Elevated Interstitial K^+^ Promotes Glycogenolysis

**Figure 4:**
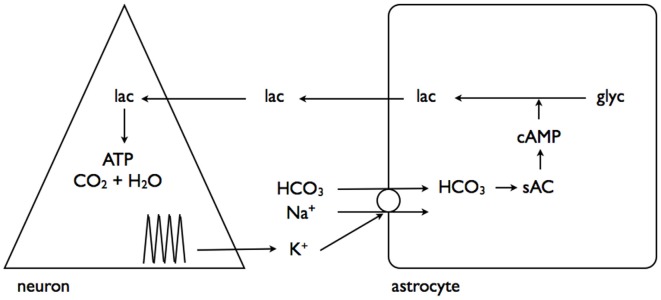
**Interstitial K^+^ stimulates astrocytic glycogenolysis.** Increased neural activity promotes elevated interstitial K^+^, which activates the Na^+^-HCO_3_^−^ coupled transporter leading to alkalization of the astrocyte cytoplasm. This activates soluble adenylyl cyclase (sAC), which in turn elevates cytoplasmic cAMP leading to glycogenolysis and transfer of lactate out of the astrocyte for neuronal oxidative metabolism. Adapted from Figure 7 (Choi et al., 2012).

A recent study has linked elevations in extracellular K^+^, as would occur during increased neuronal activity, to promotion of astrocytic glycogenolysis (Choi et al., 2012). This is a more universally applicable mechanism as all neurons/axons, be they central or peripheral, release K^+^ into the interstitium as a result of activity (Kandel et al., 2000). The greater the intensity of the activity the greater the increase in K^+^, and as demonstrated in rat optic nerve the K^+^ increase in the interstitium is buffered very efficiently by astrocytes (Ransom et al., 2000), and retreats towards baseline in the order of seconds. In the sciatic nerve the stimulus induced increases in K^+^ are attenuated, and the elevations persist for longer, suggesting less efficient buffering than occurs in central tissue (Hoppe et al., 1991). The interstitial K^+^ activates a Na^+^-HCO_3_^−^ co-transporter on the astrocyte membrane, whose activation results in intracellular alkalization. This change in intracellular HCO_3_ results in increased soluble adenylyl cyclase (sAC) activity, an enzyme that causes elevated cAMP, leading to glycogenolysis, and production of intracellular lactate (Choi et al., 2012). The lactate is transported out the cell to the neurones, where it acts as an energy substrate for oxidative phosphorylation (Figure 4).

**Figure 5:**
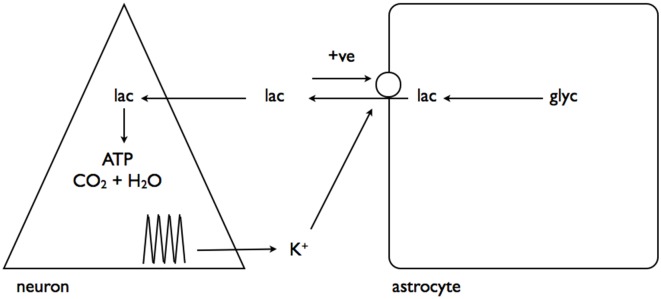
**Interstitial K^+^ promotes astrocytic lactate release via the lactate channel.** As described in Figure 4, neural activity results in elevated interstitial K^+^. This activates a channel leading to release of the cytoplasmic lactate pool into the interstitium, which, via a positive feedback loop, causes further lactate release. Adapted from Figure 8 (Sotelo-Hitschfeld et al., 2015).

In a complementary study interstitial K^+^ elevations were shown to have a separate but related effect, activating a channel on the astrocyte membrane through which pools of astrocytic lactate could flow into the interstitium, in parallel with the established route of MCTs (Sotelo-Hitschfeld et al., 2015). This is a very important route for astrocytic lactate release, since it is coupled to the membrane potential and allows lactate release against a concentration gradient, whereas the MCT is electro-neutral and net flux is governed by the trans-membrane concentrations of H^+^ and lactate (Figure 5).

## Glycogen and Memory

It is germane at this point to reflect upon the properties of glycogen and how these can confer certain metabolic advantages. Glycogen is an energy buffer, present in astrocytes that can be mobilized rapidly, without the need to expend ATP, as is the case with glucose. As such glycogen stands as a readily metabilizable energy source that would rapidly provide energy substrate. Compare this to glucose, which is delivered via the blood stream implying delivery of excess glucose would require delivery of excess blood, a process called functional hyperemia. This is a comparatively slow process (Gordon et al., 2008), and would be unable to deliver the instant energy required by neurones. This is worth bearing in mind in the following discussion of the role of glycogen during memory formation.

In studies dating back two decades the role of glycogen in supporting one of the most important brain functions, learning and memory commenced. Learning and memory are functions that can be tested in live animals via a series of standard tests (Kandel et al., 2000). The underlying cellular mechanisms are rather more difficult to pin down, but in a variety of animal models, including chick and rats, these have been ascribed to long-term potentiation (LTP) in the hippocampus. This process is quantifiable by using electrophysiological techniques, where a brief, high frequency conditioning stimulus is applied to axonal input to the CA1 pyramidal cell dendritic field. The resulting synaptic response is enhanced post-conditioning, an indication of “learning” occurring at the synapse (Malenka, 2003). The initial studies took place with the chick as the animal model. The chick was exposed to two beads, red or blue in color. The red bead was coated in an aversant compound and the chicks choice of pecking was noted. The ratio between the number of pecks of red beads and blue was measured, with an increased ratio indicative of red bead avoidance and hence learning (Hertz et al., 1996). The concentration of glycogen in the forebrain decreased concomitant with an increase in glutamate (Hertz et al., 2003). The learning process could be attenuated by inhibiting glycolysis or glycogenolysis and restored by applying exogenous aspartate and acetate, which is metabolized in the astrocytes, or application of glutamine (Gibbs et al., 2006a,b). These studies demonstrated robust learning in the chick, which appeared to involve breakdown of glycogen to ultimately produce glutamate.

The main findings of these chick experiments were extended in studies on rodents to encompass not only live animal studies on learning and memory, but investigation of the cellular mechanism underlying these processes using *in vitro* techniques. Rats were placed in a white chamber, separated from another chamber by a door. The experimenter opened the door, and when the rat entered the new chamber it received an electric footshock, and was then placed back in the white chamber. The time taken for the rat to re-enter the second chamber was taken as an index of memory, with increased latency signifying that the rat had learned to avoid the chamber (Taubenfeld et al., 2001). The rats were tested daily after the conditioning shock, and the latency to re-enter the second chamber was stable at about 300 s for up to 7 days. Injection of the GP inhibitor DAB into the rat’s brain decreased the latency indicating that memory was impaired. Exogenously dialyzed lactate into the hippocampus circumvented the inhibitory effects of DAB and augmented the latency. Electrophysiological recordings of LTP in hippocampal slices showed that in the presence of DAB the synaptic efficacy was initially enhanced, but this rapidly fell, indicative of no long-term enhancement of synaptic efficacy occurring (Suzuki et al., 2011). These data confirm a role for glycogen in the process of memory. This data appears to suggest there is a metabolic aspect to LTP, that glycogen-derived lactate is an integral component of the process (Suzuki et al., 2011). The speed with which glycogen can be metabolized makes it an ideal energy buffer, capable of rapid delivery of lactate to the neuronal elements that exhibit very rapid augmentation of synaptic efficacy in response to high frequency conditioning pulses.

This conclusion was further complemented by a study on mice where brain glycogen synthase (GS) was knocked out. Biochemical assay revealed that liver and muscle glycogen were unaffected. In these knockout animals learning and memory was impaired compared to control animals (Duran et al., 2013). Whilst initially in agreement with the previous studies, these data must be considered in the context of the role brain glycogen plays during physiological activity. The data suggests that only learning and memory were affected, since animals appeared to be normal, suggesting that brain glycogen is not vital for life, and that its absence does not lead to any significant observable pathology. Obviously more detailed studies on the effect of brain GS knockouts are required.

## Glycogen and Exercise

A role for glycogen during exercise is emerging. Its role as an energy reserve has important consequences during exercise with skeletal muscle glycogen used to provide energy. The effects, if any, of exercise on brain glycogen were unknown. Initial studies demonstrated that in rats exercising on a treadmill, the skeletal muscle glycogen fell by up to 90%, but super-compensated to ~45% after 24 h recovery. A similar scenario occurred in the brain, with a decrease of over 50%, with regional specific decreases, followed by super-compensation (Matsui et al., 2012). Interestingly with substantial exercise training the brain glycogen baseline level increased, which may be due to the increased energy demands required by the brain during exercise. However in these studies there was significant systemic hypoglycemia. Thus experiments were carried out in animals in which no hypoglycemia was achieved. In this instance brain glycogen fell, but lactate in the brain increased, implying glycogen plays a role in the brain during exercise that is not affected by hypoglycemia. Exercise results in increased serotonin turnover, which may promote glycogenolysis (Matsui et al., 2015). The same group have reported that type 2 diabetic patients had higher glycogen in the hippocampus, hypothalamus and cortex, and that MCT2 protein levels decreased, suggesting less efficient lactate shuttling (Shima et al., 2016). This effect has been correlated with exercise, which was shown to increase glycogen levels, MCT2 expression and improve cognitive performance. Thus these data support a link between glycogen levels and cognitive performance.

## Conclusion

The role(s) of nervous system glycogen are slowly emerging. Far from being a mere metabolic curiosity, it appears to act as an energy buffer, capable of the rapid delivery of the energy substrate lactate, to fuel neural function. Established occasions when this occurs are during the absence of glucose, or during hypoglycemia where glycogen derived lactate acts as a supplemental energy substrate to fuel neuronal function. As such a balance appears to be struck between ambient glucose and glycogen-derived lactate, when the former, is insufficient to meet immediate energy requirements delivery of the latter is increased. Brain glycogen also plays a pivotal role in learning and memory, where it’s presence is an absolute requirement for the transformation of short term learning into storage as memories. As such glycogen is positioned to play the key role as a buffer to supply energy substrate for short term increases in energy demand as occurs during the cellular mechanisms underlying LTP.

## Author Contributions

AMB and BRR conceived the review; edited the final version of the manuscript. AEW, LR and AMB wrote the first draft of the manuscript. AMB and BRR edited the final version of the manuscript.

## Funding

The review was funded in part by the University of Nottingham.

## Conflict of Interest Statement

The authors declare that the research was conducted in the absence of any commercial or financial relationships that could be construed as a potential conflict of interest.
